# The ATP sensitive potassium channel (K_ATP_) is a novel target for migraine drug development

**DOI:** 10.3389/fnmol.2023.1182515

**Published:** 2023-06-29

**Authors:** Amalie Clement, Sarah Louise Christensen, Inger Jansen-Olesen, Jes Olesen, Song Guo

**Affiliations:** ^1^Glostrup Research Institute, Department of Neurology, Danish Headache Center, Copenhagen University Hospital – Rigshospitalet, Copenhagen, Denmark; ^2^Department of Odontology, Panum Institute, Faculty of Health, University of Copenhagen, Copenhagen, Denmark

**Keywords:** K_ATP_ antagonist, potassium channel, migraine, headache, levcromakalim, drug target

## Abstract

Migraine is one of the leading causes of disability worldwide, affecting work and social life. It has been estimated that sales of migraine medicines will reach 12.9 billion USD in 2027. To reduce social impact, migraine treatments must improve, and the ATP-sensitive potassium (K_ATP_) channel is a promising target because of the growing evidence of its implications in the pathogenesis of migraine. Strong human data show that opening of the K_ATP_ channel using levcromakalim is the most potent headache and migraine trigger ever tested as it induces headache in almost all healthy subjects and migraine attacks in 100% of migraine sufferers. This review will address the basics of the K_ATP_ channel together with clinical and preclinical data on migraine implications. We argue that K_ATP_ channel blocking, especially the Kir6.1/SUR2B subtype, may be a target for migraine drug development, however translational issues remain. There are no human data on the closure of the K_ATP_ channel, although blocking the channel is effective in animal models of migraine. We believe there is a good likelihood that an antagonist of the Kir6.1/SUR2B subtype of the K_ATP_ channel will be effective in the treatment of migraine. The side effects of such a blocker may be an issue for clinical use, but the risk is likely only moderate. Future clinical trials of a selective Kir6.1/SUR2B blocker will answer these questions.

## 1. Introduction

Migraine is by far the most prevalent neurological disease with a lifetime prevalence of 15–20% (Stovner et al., 2018). It is sexually dimorphic with a sex ratio of 2.5 women for each man and greater severity in women. Thus, approximately 80% of the total burden of migraine is on women (Steiner et al., 2020). According to WHO migraine is number two out of all diseases causing disability and the disease has huge socioeconomic costs to society in addition to the personal costs and suffering afflicted (Abbafati et al., 2020). It is obvious that a disease of these dimensions for which no cure exists requires effective drug therapy at each acute attack and as prophylaxis in those most affected. This was illustrated by the huge success of the triptans with a peak sale at several billion USD. They are still the dominant treatment for acute migraine attacks but now mostly off patent and inexpensive. The picture was further cemented by the advent of human monoclonal antibodies against CGRP or its receptor which in a very short time has obtained impressive sales. It has been estimated that total sales of migraine medicines will reach 15.6 billion USD in 2029 (IHealthcareAnalyst I, 2023).

**Figure 1:**
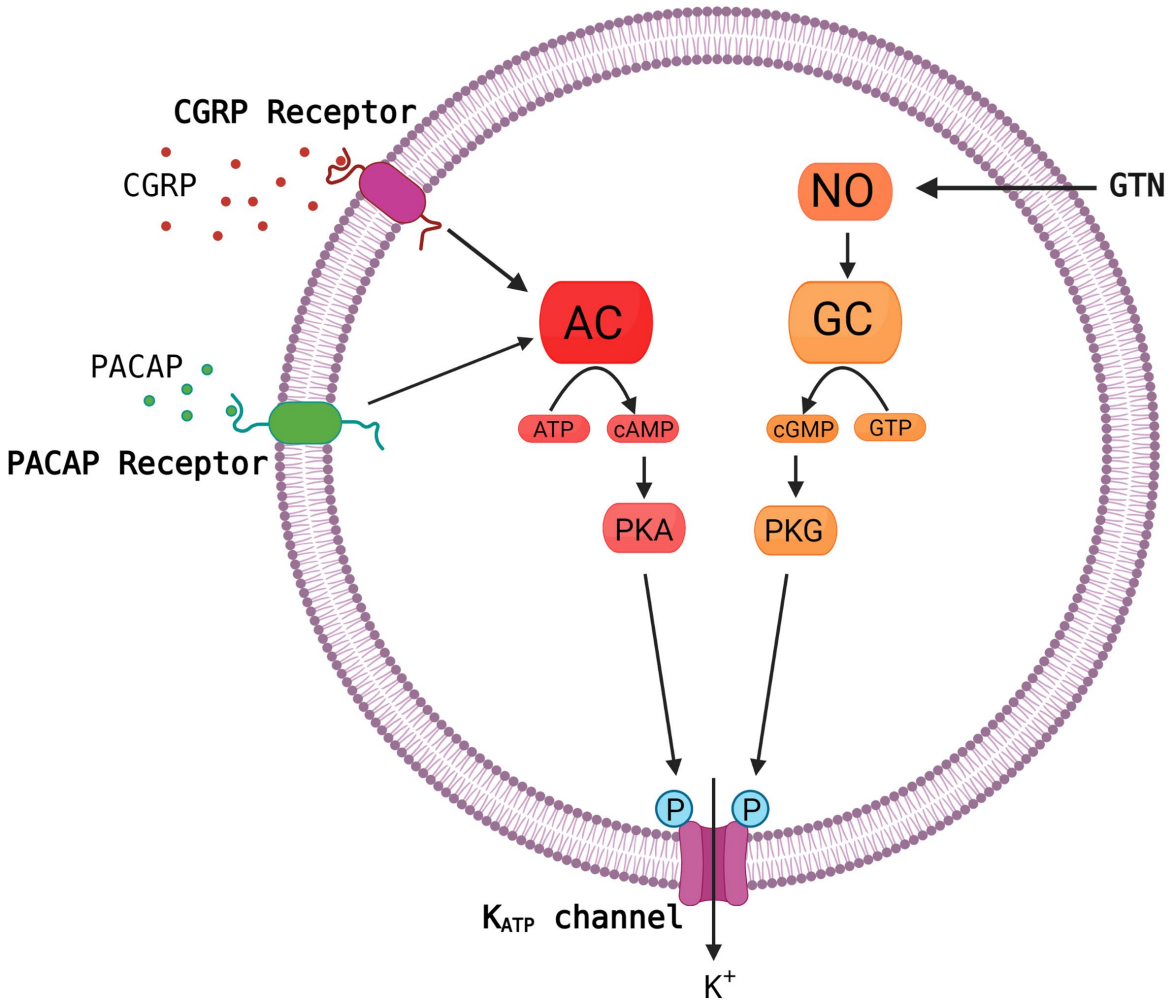
Common molecular pathways resulting in activation of the K_ATP_ channel. The migraine triggers CGRP, PACAP and nitroglycerine (GTN) are hypothesized to activate the K_ATP_ channel *via* cyclic adenosine monophosphate (cAMP) or cyclic guanosine monophosphate (cGMP) and downstream pathways. This is a simplified and hypothetical model describing the potential signaling pathways of migraine triggers and is based mainly on human experimental provocation studies (Ashina et al., 2013). More complex interactive processes and yet unknown mechanisms between signaling molecules are probably involved. AC, adenylate cyclase; NO, nitric oxide; PKA, protein kinase A; GC, guanylate cyclase; GTP, guanosine triphosphate; PKG, protein kinase G. Created with BioRender.com.

Growing evidence suggests that ATP-sensitive potassium (K_ATP_) channels are implicated in the pathogenesis of migraine as opening of the channel using levcromakalim is the most potent headache and migraine trigger ever tested. It has been suggested that the K_ATP_ channel may be the unifying mechanism for signaling pathways of established migraine triggers including calcitonin gene-related peptide (CGRP), pituitary adenylate cyclase-activating peptide-38 (PACAP38) and nitroglycerine (GTN) leading to migraine attacks (Figure 1). We have recently published a comprehensive review on the K_ATP_ channels (Clement et al., 2022), whereas this shorter review has a more general perspective on the basics of the K_ATP_ channel together with clinical and preclinical data on migraine implications. We argue that K_ATP_ channel blocking may be a novel promising target for migraine drug development.

## 2. How are migraine patients served by current drugs?

The so-called triptan wave rolled 20 years ago, and it was widely believed that the triptans had solved the migraine problem (Loder, 2010). Huge sales efforts by the pharmaceutical industry and enthusiastic doctors helped to promote this picture. Later, it turned out that the picture was not that rosy. The efficacy of triptans was originally based on rather soft success criteria, and when much stricter and more relevant criteria were applied the triptan response was much less satisfactory (Tfelt-Hansen and Olesen, 2012). A recent study showed that only 1/7 of all Danes with migraine currently uses a triptan (Davidsson et al., 2021). Whether this is due to lack of long-term efficacy or side effects is unclear, but it is not due to cost since medical service including prescriptions is free in Denmark. Thus, the treatment of migraine attacks is far from ideal. Better drugs or drugs working by a different mechanism are needed. The latter was fulfilled by the advent of small molecule CGRP receptor antagonists (Lipton and Dodick, 2004). They are less effective than the triptans but work *via* a different mechanism (Tfelt-Hansen and Loder, 2019). In prophylaxis the human antibodies against CGRP or its receptor and small molecule CGRP receptor antagonists are likewise not more effective than existing drugs but have fewer side effects than former prophylactic drugs (Charles and Pozo-Rosich, 2019; Tfelt-Hansen and Loder, 2019). Importantly, they work by a very specific mechanism and therefore have few side effects. They are also effective in non-responders to existing drugs (Ferrari et al., 2019). In conclusion, CGRP based therapies are valuable new drugs. However, these drugs are effective only in 50–60% of patients (Dodick et al., 2014a,b; Bigal et al., 2015; Sun et al., 2016) which illustrates the importance of developing more therapies with novel mechanisms of action.

## 3. Migraine heterogeneity suggests a need for precision medicine

Novel drugs such as the CGRP antagonists have been developed against unspecified migraine. In the international classification of headache disorder both the first, second and third edition (ICHD-3) subdivides migraine. The primary division is into migraine without aura and migraine with aura, but no study in recent years has focused specifically on migraine with aura which is less prevalent than migraine without aura and rarely has a high attack frequency indicating prophylactic treatment (Hauge et al., 2009). More understandably, no studies have focused on the rare sub-forms: migraine with brainstem aura, retinal migraine and familial hemiplegic migraine (Russell and Ducros, 2011). Finally, chronic migraine is the most severe form of migraine, but it represents a conglomerate of migraine without aura, migraine with aura and tension-type headache. It seems unlikely therefore, that a drug would ever be specific for chronic migraine. It has been suggested to subdivide migraine without aura and migraine with typical aura according to autonomic symptoms (Christensen et al., 2022), osmophobia (Chalmer et al., 2019), menstrual relation (Vetvik and MacGregor, 2021), genetics (Kogelman et al., 2019) and ability to sensitize during attacks (Bernstein and Burstein, 2012). But so far, there has been little correlation between these features and drug response. The huge prevalence of migraine and the known clinical and genetic heterogeneity suggest, however, that migraine in future will be best served by a multitude of drugs with a multitude of different mechanisms of action. Antagonists of the K_ATP_ channel, if effective, would be a needed addition to our current therapeutic armamentarium.

## 4. K_ATP_ channel building blocks and channel sub-types

**Figure 2:**
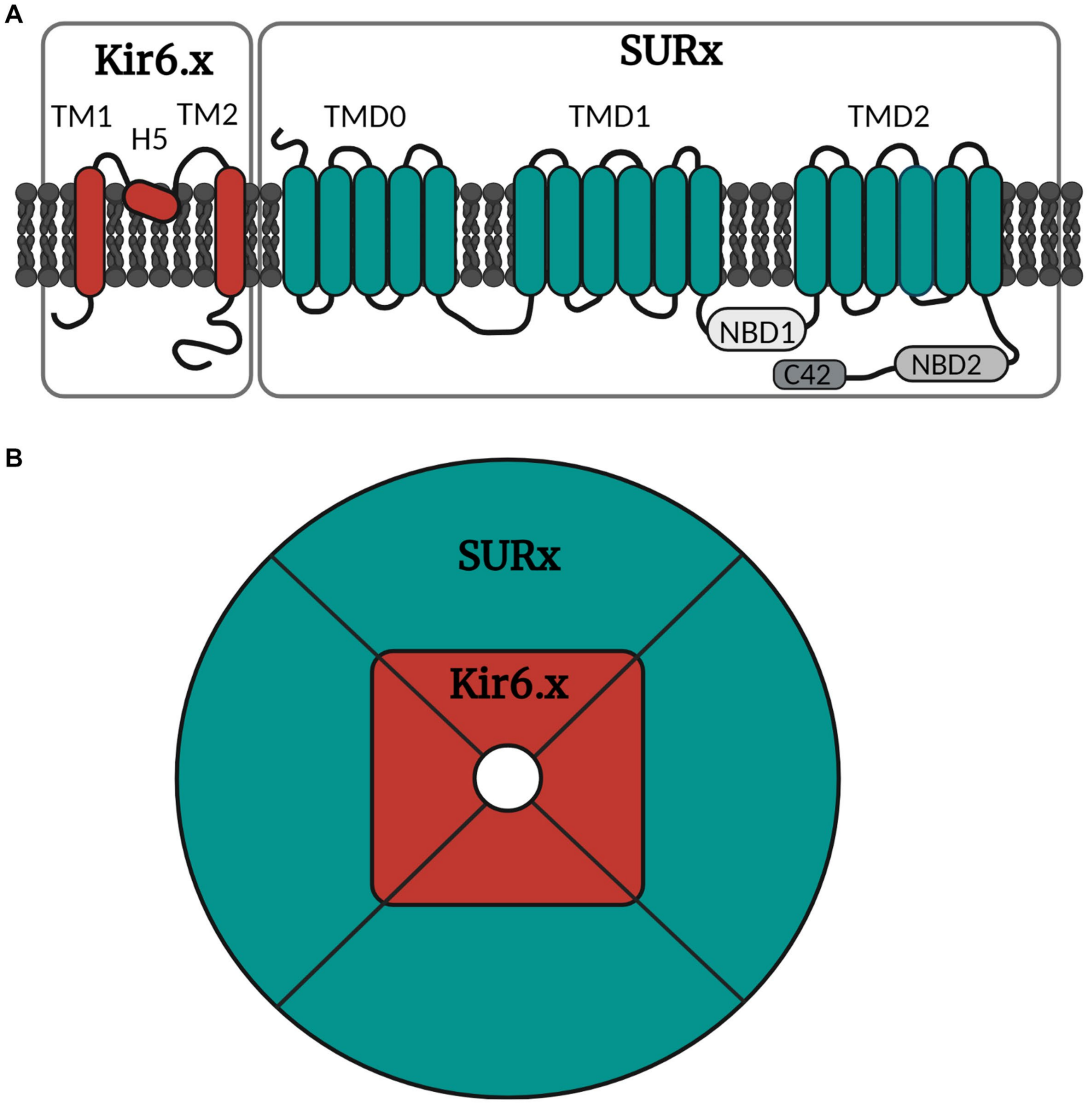
Simple illustration of the molecular and octameric K_ATP_ channel structure. **(A)** The K_ATP_ channel is arranged into subunits of Kir6.x and SURx. Kir6.x is composed of two transmembrane regions (TM1 and TM2) linked by the pore forming region H5. SURx is divided into three domains, where TMD0 consists of five transmembrane regions, while TMD1 and TMD2 consist of six transmembrane regions. SURx also holds the nucleotide binding domains (NBD1 and NBD2) and lastly the C42 C-terminal. **(B)** The channel is organized in four inner Kir6.x subunits and four outer SURx units. The Kir6.x subunits form the ion pore, while the SURx subunits form the outer structure of the channel, and these are necessary for channel activity. Created with BioRender.com.

The K_ATP_ channels belong to one of seven families of inwardly rectifying potassium channels (Kir), namely the Kir6 (Hibino et al., 2010). Kir is the building block of the ion pore which consists of an aggregation of four units (Figure 2). The Kir6 subfamily is divided into Kir6.1 and Kir6.2, and around the ion pore is the modifying unit sulfonylurea receptor called SUR, which exists in three sub-forms, SUR1, SUR2A and SUR2B (Babenko et al., 1998). Thus, many configurations of the K_ATP_ channel can exist depending on the combination of inner and outer building blocks. Drugs can either target the SUR unit or the pore itself. The distribution of the different types of K_ATP_ channels in different tissues is shown in Table 1.

**Table 1:** Subunit composition and tissue expression of K_ATP_ channels in different species.

Channel structure	Tissue	Species	References
Kir6.1/SUR1	Retina	Frog + tadpole	Skatchkov et al. (2002)
	Nervous system	Frog	Eaton et al. (2002)
Kir6.1/SUR2B	Vascular smooth muscle	Human + pig	Ploug et al. (2008)
		Rat	Ploug et al. (2006), Ploug et al. (2010), and Li et al. (2003)
		Mouse	Christensen et al. (2022) and Yamada et al. (1997)
	Connective tissue	Mouse	Christensen et al. (2022)
	Conduction system of the heart	Mouse	Bao et al. (2011)
Kir6.2/SUR1	Pancreas	Rat	Ploug et al. (2010)
		Mouse	Isomoto et al. (1996)
		Dog	Donley et al. (2005)
	Heart	Rat	Ploug et al. (2010)
		Mouse	Isomoto et al. (1996) and Flagg et al. (2008)
	Nervous system	Rat	Zoga et al. (2010) and Ploug et al. (2010)
	Skeletal muscle	Mouse	Mele et al. (2014)
Kir6.2/SUR2A	Heart	Mouse	Bao et al. (2011), Isomoto et al. (1996), and Flagg et al. (2008)
	Skeletal muscle	Rat	Inagaki et al. (1996)
Kir6.2/SUR2B	Non-vascular smooth muscle	Mouse	Isomoto et al. (1996) and Koh et al. (1998)
	Nervous system	Rat	Zoga et al. (2010)
	Conduction system of the heart	Mouse	Bao et al. (2011)
	Skeletal muscle	Rat	Inagaki et al. (1996)
		Mouse	Mele et al. (2014)

## 5. K_ATP_ channel agonists in other diseases cause headache

Several K_ATP_ channel agonists have been developed for clinical practice including levcromakalim, nicorandil, tifenazoxide, pinacidil, minoxidil, diazoxide and with indications for asthma, ischemic heart disease, hypertension, hair loss and hypoglycaemia. Except for nicorandil, none of these drugs is widely used in clinical practice but headache is reported as a common side effect for all these K_ATP_ agonists probably due to their vasodilating effects of cranial arteries (Table 2; Jahangir and Terzic, 2005). Notably, the most frequent side effect reported for levcromakalim, nicorandil and pinacidil treatment was headache (Kidney et al., 1993) - mostly as mild to moderate in intensity that generally occurred early in treatment. Headache was also often responsible for patients discontinuing their treatment (Simpson and Wellington, 2004). Altogether, these findings sparked curiosity in investigating the role of K_ATP_ channels in basic science and human experimental models of migraine.

**Table 2:** Headache incidences registered during clinical trials with K_ATP_ channel agonists.

K_ATP_ agonist	Study design	Indication	Headache	Ref.
Levcromakalim	RCT	Asthma	76%	Kidney et al. (1993)
Nicorandil	Review (4 RCTs)	Ischaemic heart disease	36%	Simpson and Wellington (2004)
Tifenazoxide (NN414)	RCT	Healthy subjects	Up to 33% depending on dose	Zdravkovic et al. (2007)
Pinacidil	RCT	Hypertension	21%	Goldberg (1988)
Minoxidil	Open label	Hypertension and hair loss	21%	Sanabria et al. (2022)
Diazoxide	RCT	Hypoglycaemia	Yes (frequency not reported)	Product Information (2015)

## 6. Cephalic localization and function of K_ATP_ channels (*in vitro*)

### 6.1. Cranio-vascular

In intracranial arteries from rat, pig and human Kir6.1 and SUR2B mRNA and protein are predominantly expressed. Only low levels of Kir6.2 and SUR1 and no SUR2A are detected (Ploug et al., 2006). The subdivided location of K_ATP_ channels in cephalic arterial smooth muscle cells and endothelial cells have not yet been examined. However, K_ATP_ channels in endothelial and smooth muscle cells of peripheral arteries are of the Kir6.1/SUR2B subtype (Aziz et al., 2017; Li et al., 2020). Activation of K_ATP_ channels leads to hyperpolarization of smooth muscle cells (Brayden, 2002) that prevents the opening of depolarization activated Ca^2+^ channels. This causes a decrease in Ca^2+^ entry to the cell leading to vasodilatation (Quast, 1996). In endothelial cells activation of K_ATP_ channels elevate intracellular calcium by inducing hyperpolarization and augmenting the driving force for potential dependent Ca^2+^ influx. In this way, the K_ATP_ channel agonist may promote Ca^2+^-dependent formation of endothelium-derived relaxing factors such as nitric oxide (Lückhoff and Busse, 1990).

Pharmacological *ex vivo* and *in vivo* studies show vasodilatory effects of K_ATP_ channel openers in rat and pig intracranial arteries. These responses are inhibited by high doses of the non-selective K_ATP_ channel blocker glibenclamide and lower doses of the Kir6.1 blocker PNU-37883A supporting involvement of Kir6.1/SUR2B K_ATP_ channels in cranio-vascular responses (Gozalov et al., 2005; Ploug et al., 2006, 2008). Removal of rat basilar artery endothelium slightly but significantly, inhibited K_ATP_ channel opener induced vasodilation, suggesting a partial involvement of endothelial K_ATP_ channels (Jansen-Olesen et al., 2005). *In vivo* studies have shown CGRP to activate K_ATP_ channels and thereby participate in CGRP mediated dilation of rat meningeal arteries (Gozalov et al., 2008). Interestingly, glibenclamide attenuated this CGRP-induced vasodilation *in vivo* but was without effect *ex vivo* (Gozalov et al., 2008).

### 6.2. Neuronal

In the nervous system, K_ATP_ channels are highly expressed in neurons, where they cause hyperpolarization resulting in reduced excitability, potassium efflux (Babenko et al., 1998; Seino and Miki, 2003; Hibino et al., 2010) and consequently reduced neurotransmitter release (Soundarapandian et al., 2007; Yildirim et al., 2021; Clement et al., 2022). Furthermore, K_ATP_ channels of the nervous system are suggested to mediate neuroprotection in different scenarios like hypoxia and oxidative stress (Zoga et al., 2010; Country and Jonz, 2021; Clement et al., 2022). Especially the mitochondrial K_ATP_ channels have been highlighted in neuroprotection during excitatory toxicity stimulation (Soundarapandian et al., 2007), as activation of mitochondrial K_ATP_ channels cause depolarization and reduces neuronal death probably by decreasing influx of Ca^2+^ which lowers mitochondrial Ca^2+^ levels (Soundarapandian et al., 2007). This process hinders loss of oxidative phosphorylation and ATP depletion (Country and Jonz, 2021; Clement et al., 2022), but overall the mechanisms underlying the mitochondrial K_ATP_ channel-related neuroprotective effects remain unclear.

K_ATP_ channels have been found on nerve terminals of rat hippocampal slices, likely on both noradrenergic and glutamatergic nerve endings (Freiman et al., 2001). Likewise, K_ATP_ channels are found on nerve terminals prepared from rat motor cortex (Lee et al., 1995). K_ATP_ channels (Kir6.2/SUR1 and Kir6.2/SUR2) were also identified on neurons, glial satellite cells and Schwann cells of dorsal root ganglion isolated from rat (Zoga et al., 2010). In this study, K_ATP_ channel expression was reduced after painful axotomy, suggesting an involvement in pain perception and a potential target for therapeutic intervention.

Lastly, both Kir6.1 and Kir6.2 expression (using RT-PCR studies) are found in pure glial cultures of rat midbrain (Toulorge et al., 2010). Interestingly, expression of K_ATP_ channels was not found on the migraine relevant trigeminal ganglia isolated from mouse, using both RT-PCR and Western blotting, but high expression on dura mater and brain arteries (Christensen et al., 2022). This pattern is supported by RNAseq data from human and mouse trigeminal ganglion (Yang et al., 2022).

## 7. Human K_ATP_ agonist and antagonist studies

Both levcromakalim (K_ATP_ channel agonist) and glibenclamide (K_ATP_ channel blocker) have been tested in human experimental studies of headache.

Intravenous infusion of levcromakalim induced migraine attacks in 100% of migraine patients without aura (*n* = 16) making it the most potent migraine-triggering compound to date ever tested (Al-Karagholi et al., 2019). The provoked headache developed very quickly with a median time to onset of 20 min after infusion, and for that reason, no prodrome symptoms were registered. Interestingly, when levcromakalim was given to migraine patients with visual aura (*n* = 17), 59% developed migraine attacks with visual aura (Al-Karagholi et al., 2021). These findings suggest that levcromakalim is a migraine aura-inducing compound and that the K_ATP_ channel may play a role in both migraine aura and migraine headache. It has been argued that levcromakalim most likely induces visual aura and migraine headache through distinct mechanisms, because it very potently triggers migraine without aura, and no aura symptoms, in migraine patients who have never experienced aura. Also, levcromakalim was also able to induce migraine without aura in some patients who previously have only experienced visual aura symptoms not accompanied by headache or migraine (Al-Karagholi et al., 2021). A possible underlying mechanism may be that levcromakalim increases extracellular potassium concentrations in neurons, glial cells, and brain vasculature, which depolarizes neighboring cells thereby triggering a wave of cortical spreading depression (CSD) underlying the visual aura. Nevertheless, it is unclear whether levcromakalim can cross the blood–brain barrier (BBB) but based on its small molecular weight (286.33 Da) and probable lipophilic properties, a central effect cannot be ruled out.

In healthy volunteers (*n* = 14), levcromakalim induced headache in 86% of participants and dilated the middle meningeal arteries (MMA). The dilatation was reversed by sumatriptan injection (Al-Karagholi et al., 2019). Notably, 14% of the healthy participants reported migraine-like attacks after levcromakalim. Also, the headache-associated dilatation of MMA is noteworthy, as the MMA is the only cranial artery with dilation on the pain side during the early phase of experimentally induced migraine attacks (Khan et al., 2019). However, intradermal and intramuscular levcromakalim injections did not produce more pain than placebo (Al-Karagholi et al., 2019) indicating that levcromakalim-induced headache pain is unlikely to be the result of direct activation of peripheral nociceptors.

Glibenclamide is a non-specific K_ATP_ channel blocker widely used for the treatment of diabetes mellitus type 2 to increase insulin secretion. It belongs to the second generation of sulfonylureas (Gribble and Reimann, 2003; Mannhold, 2004). It is yet unknown whether glibenclamide can prevent migraine attacks in adults with migraine. Nevertheless, in healthy volunteers, pre-treatment with glibenclamide did not prevent levcromakalim-induced headache (Al-Karagholi et al., 2020) and did not attenuate levcromakalim-induced vascular changes, e.g., mean global CBF, intracranial artery circumferences, mean arterial blood pressure and heart rate (Al-Karagholi et al., 2021). However, glibenclamide seemingly delayed the onset of levcromakalim-induced headache with median time to headache onset 180 min (Al-Karagholi et al., 2020) versus 20 min (Al-Karagholi et al., 2019). As previously mentioned, glibenclamide is a non-selective K_ATP_ channel blocker with a higher affinity for the SUR1 subunit compared to the SUR2 subunits. The K_ATP_ channel opener levcromakalim which is one of the most effective drugs to induce headache in humans, has a high affinity to the SUR2B subtype. The high expression of SUR1 in pancreas mediate an increase in insulin secretion which limits the maximum tolerated dose of glibenclamide in humans due to severe risk of hypoglycaemia. Although the blood glucose levels were stabilised by infusion of glucose the dose of glibenclamide could still be too low to inhibit the SUR2B subtype of the K_ATP_ channel upon which levcromakalim acts. Glibenclamide did not prevent CGRP- and PACAP-induced headache and hemodynamic changes in healthy volunteers (Coskun et al., 2021; Kokoti et al., 2022). Collectively, these findings imply that glibenclamide does not inhibit the headache-inducing effects of K_ATP_ channel activation, probably because glibenclamide is non-specific and primarily inhibits the SUR1 subunit of the K_ATP_ channel. Thus, more selective K_ATP_ channel blockers selective for the Kir6.1/SUR2B subtype are needed to further examine the therapeutic potential of K_ATP_ channel inhibitors in migraine.

## 8. Preclinical K_ATP_ channel agonist and antagonist studies

A range of migraine triggering substances identified in studies of human experimental migraine have also been studied in rodent models where they induce a state of hypersensitivity to various sensory stimuli (Bates et al., 2010; Pradhan et al., 2014; Rea et al., 2018; Demartini et al., 2019; Christensen et al., 2021; Kuburas et al., 2021; Ernstsen et al., 2022). Repeated systemic administration of levcromakalim induced hypersensitivity to tactile stimulation with von Frey filaments (Christensen et al., 2020, 2021; Wu et al., 2022), heat (Christensen et al., 2022), and increased c-Fos expression in the spinal trigeminal nucleus (Wu et al., 2022). In contrast, local intraplantar and intracerebroventricular administration did not lower sensory threshold (Wu et al., 2011; Christensen et al., 2022).

Tactile hypersensitivity induced by migraine triggers GTN, cilostazol, levcromakalim and PACAP38 was fully or partially prevented by pre-administration of glibenclamide (Christensen et al., 2020, 2021; Ernstsen et al., 2022). In a distinct rat model of migraine presenting with spontaneous (inheritable) hypersensitivity in cephalic dermatomes (Oshinsky et al., 2012; Munro et al., 2018), both glibenclamide and gliquidone treatment normalized cephalic sensitivity thresholds, but did not increase the otherwise normal hind-paw threshold (Christensen et al., 2020). These findings initiated further investigation of K_ATP_ channel subtype specificity. Significant contribution of the vascular Kir6.1/SUR2B channel subtype to migraine pain generation was indicated as mice lacking Kir6.1 specifically in smooth muscle cells did not sensitize to the same degree as controls following repeated dosing of neither GTN nor levcromakalim (Christensen et al., 2022). Although, vasodilation seems to play a role in the levcromakalim-induced hypersensitivity, it alone may not explain it. Other unclear non-vascular mechanisms are likely to be at play as well. Because glibenclamide in high doses only partially blocked the hypersensitivity induced by PACAP38, whereas the relatively low dose of glibenclamide inhibited levcromakalim-induced hypersensitivity in rodents without affecting the vasodilation produced by levcromakalim *in vivo* and *ex-vivo* (Christensen et al., 2020, 2021; Ernstsen et al., 2022). Also, the levcromakalim-induced hypersensitivity can be abolished by CGRP-antagonizing drugs, suggesting that levcromakalim causes CGRP release *via* an unknown mechanism. The CGRP release may also not be secondary to vasodilation produced by K_ATP_ activation because the vasodilation is not inhibited by glibenclamide at concentrations that inhibit the hypersensitivity. In *ex vivo* organ preparations of dura mater and trigeminal ganglion glibenclamide inhibited capsaicin induced CGRP release (Christensen et al., 2020) which also supports the fact that K_ATP_ channel blockers may have a positive effect on migraine *via* non-vascular mechanisms. Opening of K_ATP_ channels does not directly cause CGRP release in the isolated tissue (Ploug et al., 2012; Christensen et al., 2021).

## 9. Likelihood of effectiveness of K_ATP_ antagonists

There are many examples of therapeutic efficacy of antagonists of migraine provocation (Ashina et al., 2017). Angiography provoked attacks of migraine with aura and studies of regional cerebral blood flow demonstrated that cortical spreading depression is the likely mechanism of the aura (Olesen et al., 1981, 1990). A blocker of cortical spreading depression, tonabersat, was effective in the prophylaxis of migraine with aura (Hauge et al., 2009). GTN, *via* liberation of nitric oxide, was effective in provoking migraine without aura attacks and the non-selective blocker of nitric oxide synthases L-NMMA was effective in treating acute attacks of migraine without aura (Lassen et al., 1997). Finally, and most convincingly, CGRP was effective in inducing migraine attacks without aura in patients (Lassen et al., 2002) and this was crucial for developing brain non-penetrant small molecule CGRP receptor antagonists as well as non-penetrant human monoclonal antibodies against CGRP or its receptor. Antagonism to a migraine provoking substance is therefore often predictive of clinical efficacy but not always. Histamine is equally effective to nitroglycerin in inducing migraine attacks (Lassen et al., 1995), but antihistamines have been on the market for more the 50 years and have received several trials in migraine without convincing results (Lassen et al., 1996; Vollesen et al., 2019). It makes sense that migraine can be experimentally induced by mechanisms that also occur during spontaneous attacks but, likewise, it can be induced by mechanisms that are not part of the mechanisms of spontaneous migraine attacks. Therefore, it cannot be concluded that the strong migraine-provoking effect of levcromakalim in adults with migraine proofs that an antagonist of the K_ATP_ channel will effectively prevent or resolve migraine attacks. However, we believe it to be likely. But there are other caveats. K_ATP_ channels are abundant in the heart and knockout of specific K_ATP_ subfamilies in rodent models have been lethal in mice (Miki et al., 2002). To date, there are no studies of a selective Kir6.1/SUR2B knockout model, which is less abundant in the heart. If opening of the channel is important for migraine attack development, then the opening would primarily be in K_ATP_ channel subtypes localized in the cephalic vasculature or other cephalic structures and therefore a selective blocker would selectively affect these structures and not the heart.

## 10. Conclusion

K_ATP_ channels are acknowledged as viable therapeutic targets for migraine treatment as human data show that opening of the K_ATP_ channel induces headache in healthy subjects and migraine in migraine sufferers. Still, drug discovery remains a formidable obstacle. Currently, only data from migraine rodent models has illustrated a migraine-relevant effect of blocking the K_ATP_ channel activity. To move forward, additional research must be conducted on the specific subtypes of the K_ATP_ channel to gain a deeper comprehension of their structures, functions, and distribution for the development of more selective and effective drugs. We believe there is a likelihood that an antagonist of the Kir6.1/SUR2B sub-type will be effective in migraine. Only development and clinical testing of a future selective K_ATP_ channel blocker with attention on side effects can answer these questions.

## Author contributions

All authors listed have made a substantial, direct, and intellectual contribution to the work, and approved it for publication.

## Funding

SG is supported by the BRIDGE – Translational Excellence Programme (bridge.ku.dk) at the Faculty of Health and Medical Sciences, University of Copenhagen, funded by the Novo Nordisk Foundation. Grant agreement no. NNF20SA0064340 (2021 fellows). Also, this work was supported by Candys Foundation.

## Conflict of interest

JO owns shares in the start-up company Cephagenix.

The remaining authors declare that the research was conducted in the absence of any commercial or financial relationships that could be construed as a potential conflict of interest.

## Publisher’s note

All claims expressed in this article are solely those of the authors and do not necessarily represent those of their affiliated organizations, or those of the publisher, the editors and the reviewers. Any product that may be evaluated in this article, or claim that may be made by its manufacturer, is not guaranteed or endorsed by the publisher.
